# Effects of hydrokinesitherapy on balance and walking ability in stroke survivors: a systematic review and meta-analysis of randomized controlled studies

**DOI:** 10.1186/s11556-019-0227-0

**Published:** 2019-11-13

**Authors:** Guanli Xie, Tao Wang, Bo Jiang, Yan Su, Xiaoxia Tang, Ying Guo, Jianglong Liao

**Affiliations:** 1 Yunnan University of Chinese Medicine, Kunming, Yunnan China; 2 Kunming Municipal Hospital of Traditional Chinese Medicine, 2628 Xianyuan Road, Cheng Gong District, Kunming, 650500 Yunnan China; 3 Kunming Municipal Hospital of Traditional Chinese Medicine and the Jiang Bo Famous Medical Studio, Kunming, Yunnan China; 4 Kunming Combination of Chinese and Western Medicine Minimally Invasive Spine Technology Center, Kunming, Yunnan China; 5 Kunming Municipal Hospital of Traditional Chinese Medicine, 223 Guanxing Road, Guan Du District, Kunming, 650200 Yunnan China

**Keywords:** Stroke, Hydrokinesitherapy, Balance, Walking ability, Meta-analysis

## Abstract

**Background:**

Balance and walking impairment are common dysfunctions after stroke. Emerging data has demonstrated that hydrokinesitherapy may have a positive influence on improvement of balance and walking ability. However, there is no firm evidence to support these results. Therefore, the aim of this review is to evaluate the effects of hydrokinesitherapy in stroke survivors systematically.

**Methods:**

Medline, EMBASE, Cochrane Central Register of Controlled Trials (CENTRAL) in the Cochrane Library, CINAHL and SPORTDiscus were systemic searched from their inception to Septemter 30, 2018. RevMan 5.3 software was used to perform data synthesis. The fixed-effect model or random-effect model was employed according to the results of heterogeneity test. The mean differences (MD) or standardized mean difference (SMD) was used to evaluate the pooled effect of hydrokinesitherapy on balance function, walking ability and activty of daily life (ADL).

**Results:**

A total of 13 studies were included involving 381 stroke survivors. Meta-analysis results indicated that hydrokinesitherapy could improve balance ability based on three test: Berg balance scale (BBS: MD = 3.84, 95% confidence interval (95% CI) 2.84 to 4.86, *P* < 0.001), Time Up To Go Test (TUGT: MD = − 1.22, 95% CI − 2.25 to − 0.18, *P* = 0.02, fixed-effect model), Functional Reach Test (FRT: MD = 2.41, 95% CI 1.49 to 3.33, *P* < 0.001). Additionally, we found a weakly positive effect on walking speed (SMD = 0.75, 95% CI 0.26 to 1.25, *P* = 0.003) and walking ability test (SMD = 0.36, 95% CI 0.04 to 0.68, *P* = 0.03). There was no significant difference between experimental group and control group in terms of ADL.

**Short conclusion:**

Hydrokinesitherapy can improve balance function and had a weakly positive effect on walking ability in stroke survivors. We did not find sufficient evidence to indicate that hydrokinesitherapy could improve the ADL of stroke survivors. However, due to the methodological shortcoming and small number of included studies, caution is needed when interpreting these results. Due to imprecision and publication bias, the quality of the evidence was downgraded to “low-quality” for the primary outcomes of balance and walking ability.

**Trial registration:**

CRD42018110787.

## Introduction

Stroke, a lack of blood supply to the brain, is the third leading cause of chronic disability in adults worldwide [1, 2]. It was reported that there are 16.9 million patients with initial stroke, 33 million stroke survivors, 5.9 million stroke-related deaths, and 102 million Disability-Adjusted Life Years (DALYs) lost, with most of the burden in low-income and middle-income countries according to Global Burden of Disease (GBD) Study 2010 [1]. Stroke survivors suffer varying functional disorder including balance dysfunction, walking disorder, cognitive impairment [3]. Of all the functional disorders caused by stroke, balance impairment is the most common and considered to be primary impairment after stroke [4–8]. Ambulatory impairment is another devastating sequelae as more than 30% of survivors remain unable to walk independently 6 months after a stroke [3, 9–11]. Balance dysfunction and walking impairment make it difficult to complete activities of daily life (ADLs) safely, move at home or in the community, and live independently [6, 7]. Meanwhile, balance impairment and walking dysfunction can result in lower confidence for movement and in completing ADLs, which may in turn further reduce activity [12]. If not detected or left untreated, balance and walking impairment can lead to a cascade of serious, undesirable, and expensive events [13, 14]. Balance and walking ability were also significant predictors of functional improvement after stroke [15]. Thus, therapeutic intervention strategies need to pay close attention to improving balance and walking ability [16].

Various rehabilitation programs have been developed to solve such problems as balance dysfunction, walking impairment, and limited ADLs related to stroke [3]. These programs include Brunnstrom approaches, neurodevelopmental treatment (NDT), proprioceptive neuromuscular facilitation, and motor relearning programs [17, 18]. Although these rehabilitation programs have been shown to be beneficial for stroke survivors, no superior specific approach or program has been discovered [3], as other effective rehabilitation programs are being developed [19]. However, only approximately 5 to 20% of post-stroke survivors achieve complete functional recovery after intensive rehabilitation efforts [20, 21]. Therefore, there is an urgent need for new appropriate and promising rehabilitative program for inpatients and outpatients with stroke.

Hydrokinesitherapy, a promising intervention, combines the revitalizing and strengthening effects of the physical exercise with the muscle-relaxation and physical properties of water submersion [22]. This technique was developed some decades ago when it was used as a rehabilitative approach for poliomyelitis [23]. It was demonstrated that this treatment could be used as a rehabilitation program for patients with “weak muscles”. Later studies have shown that hydrokinesitherapy was beneficial for fitness and strength maintenance in various chronic conditions, including multiple sclerosis [24, 25], Parkinson’s disease (PD) [26], spinal cord injury [23], cerebral palsy [27], and arthritis [28]. Hydrokinesitherapy has also been demonstrated to be suitable and safe for patients with stroke [29]. Remarkably, recent studies suggested that hydrokinesitherapy has positive effects on posture balance control [30], muscle strength [31], fitness [32], anxiety and depression scores [33] and ADLs [34] in patients with stroke.

Hydrokinesitherapy has positive benefits for stroke patients thanks to the essential physical properties of water such as its favorable specific gravity, buoyancy, density, hydrostatic pressure, cohesion and viscosity [22, 35]. As the body is gradually immersed in water, buoyancy is created, progressively offloading immersed joints. It was reported that when the water depth is equal to the symphysis pubis, 40% of body weight is effectively offloaded, and if the depth is equal to the umbilicus or xiphoid, offloading increase to approximately 50% or 60% respectively [35]. Thus, compared with land-based exercise, hydrokinesitherapy enables stroke survivors to support their body weigh more easily. It also allows patients to increase the active range of motion activities without causing pain, as well as strength and even gait training which present further difficulties on land [35]. Furthermore, hydrokinesitherapy allows patients to enhance control of strengthening activities comfortably due to the viscosity of water [36].

Another positive effect of water is the relaxation effect and the pain perception effect [35]. Hydrokinesitherapy can inhibit the sensitivity of muscle spindles and skin. This results in a reduction of gamma fiber activity with a consequent reduction of muscle spasm and contractures, leading to remission of pain and muscle relaxant effects [37]. All of these effects make water-based exercises a promising therapeutic intervention when targeting balance and walking ability improvement for patients after stroke.

A previous Cochrane review demonstrated that there is no firm evidence to support or refute the effectvieness of hydrokinesitherapy on ADLs for stroke patients [29]. Multiple clinical studies have been reported since that systematic review was published. Emerging data has demonstrated that hydrokinesitherapy rehabilitation programs may have a positive influence on improvement of balance ability [30, 38–40], muscle strength [31, 38, 41], functional walking ability [42], cardiovascular fitness [32, 34], ADL and impaired mental health [33] of stroke survivors. There is also study focused on this topic in progress [43]. As a consequence of such, further evidence, the safety and clinical effectiveness of hydrokinesitherapy for patients with stroke should be reevaluated to provide further clinical evidence both for clinicians and patients. Based on all of above, the aim of this review is to elucidate whether hydrokinesitherapy rehabilitation programs are safe and beneficial to improve balance and walking ability for stroke survivors.

## Main text

### Methods

#### Trial registration

The design of this systematic review followed the guidelines of the Cochrane Collaboration. The protocol of this systematic review was registered at the International Prospective Register of Systematic Review, PROSPERO, and published prior to this study [44].

#### Search strategy

Online electronic databases, including Medline (via PubMed), EMBASE (via embase.com), Cochrane Central Register of Controlled Trials (CENTRAL) in the Cochrane Library, CINAHL (via EBSCO host) and SPORTDiscus (via EBSCO host) were systematic searched from their inception to September 30, 2018 without language restrictions. Relevant keywords related to hydrokinesitherapy as MeSH terms and free words (e.g. “hydrotherapy” or “aquatic exercise”, or “water-based exercise”) were used in association with MeSH terms and free words of stroke (e.g. “stroke” or “cerebrovascular disorders” or “hemorrhage”). Additional trials were identified through the references list of retrieved articles. Full search strategy for each database is available online in Additional file 1 and the search strage used for Medline (via Pubmed) was reported in protocol of this study [44].

#### Study eligibility criteria

The studies included must have met the inclusion criteria as follows: (1) Participants: patients who had suffered hemorrhagic or ischemic stroke regardless of age and gender. Stroke must have been diagnosed according to the World Health Organization (WHO) definition [45], or a clinical definition of stroke when the WHO definition was not specifically stated, or confirmed by CT or MRI. The initial level of impairment and the duration of stroke were not limited. (2) Intervention: any single or collective intervention involving structured treatment of participants in water that is designed and reproducible. Intervention must have been carried out by a well-trained health care professional rather than using a spa pool or bathtub. The duration and frequency of hydrokinesitherapy were not limited. (3) Control: the control group received non-water-based exercise or no treatment. (4) Outcomes were not limited and (5) Study design was limited to clinical randomized controlled trials (RCTs). Only studies that have full-text articles in English were included in this study in order to ensure the accuracy of data extraction.

#### Study selection and data extraction

Systematic search, selection of studies and data extraction were performed between October 2018 and December 2018. All records were imported into a reference management software (Endnote X7) and then duplicate records from the same trial were removed. Potentially eligible studies and obviously irrelevant reports were identified by two reviewers (GLX and TW) independently. Potential full texts studies were obtained according to inclusion criteria. Any disagreement was resolved by discussion and a final decision was determined in consultation with a third author (JLL), if necessary.

Data were extracted from the included studies by one reviewer (GLX) using a purpose-designed form and verified by another review author (TW). The extracted data contained basic information about the publications (title of article, authors, year of publication, title of periodical, etc), design (randomization, allocation concealment, and blinding, etc), patient characteristics (number, age, gander, inclusion/exclusion criteria, etc), interventions (type of intervention, frequency, intensity and duration for each group) and outcomes (measurement instruments, follow-up, drop-out and adverse events, etc) were extracted from each study. When missing data was observed, we contacted original author of trial in an effort to collect further data. In order to meet the needs of data conversions (e.g. mean to mean difference), Miscrosoft Excel was used to manage extracted data before entry into RevMan 5.3. Any disagreement regarding data extraction were resolved via discussion and a final decision was determined in consultation with a third author (YG) if any disagreement persisted.

#### Assessment methodological quality of included studies

Two independent reviewers (GLX and TW) assessed the methodological quality of the included studies using the “Risk of Bias” table recommended by the Cochrane Handbook for Systematic Reviews of Interventions [46]. Six domains involving seven items include selection bias (random sequence generation and allocation concealment), performance bias (blinding of particpants and personnel), detection bias (blinding of outcome assessment), attrition bias (incomplete outcome data), reporting bias (selective reporting) and other biases. Other sources of bias affecting precision of an estimate include small sample size, different length of follow-up, stopped early due to some data-dependent process, extreme baseline imbalance, or other problems. Each item for each study was categorized as “high risk”, “unclear” and “low risk” depending on the level of bias. For the item “blinding of participants and personnel (performance bias)”, all the included studies in this review were considered to be of “high risk” because it was impossible to blind the intervenor and participants during hydrokinesitherapy rehabilitation program. Any discrepancies were resolved via discussing and consulting with a third reviewer (JLL).

#### Data analysis

Cochrane Review Manager (RevMan 5.3) was used to perform data analysis. Mean difference (MD) or standardized MD (SMD) with 95% confidence intervals (95% CIs) were employed to measure trial outcomes for continuous data. If data were available and heterogeneity test detected no significant heterogeneity among included studies, the fixed-effect model was employed to estimate pooled effect; otherwise, the random-effect model was utilized. Chi-squre test and Higgins I^2^ value were used to analyze statistical heterogeneity. A Chi-squre test results of *P* < 0.1 with I^2^ > 75% represented significant statistical heterogeneity. Subgroup analysis was conducted to investigate the clinical heterogeneity according to the different measure instruments. Sensitivity analysis was employed to evaluate the stability of summary effect size. The two-sided level of 0.05 was considered to show significance in the meta-analysis.

#### Grading the quality of evidence

The Grading of Recommendations Assessment, Development and Evaluation (GRADE) recommended classification method was used to evaluate quality of evidence based on risk of bias, inconsistency, imprecision, publication bias and indirectness.

## Results

### Study identification

**Fig. 1:**
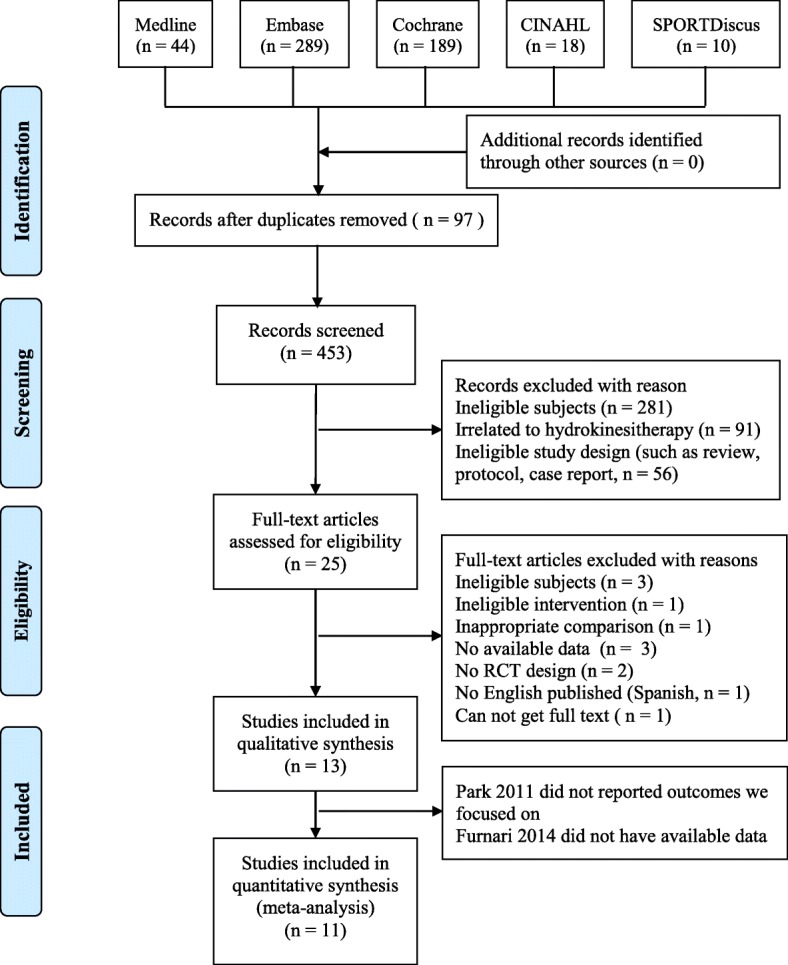
Flow diagram of the study selection process

In total, 550 articles were identified from online databases. A total of 79 records were identified as duplicate reports and 428 records were considered to be irrelevant records by two reviewers based on abstracts and titles. Further, twelve full texts were excluded because they did not meet the inclusion criteria. The remaining 13 RCT full text trials were included in this systematic review based on the inclusion criteria. However, one study did not provide the data needed and one study did not report the outcomes we focused on. We attempted to contact original authors by email but we did not receive a responses. Thus, we did not included these two studies in our meta analysis but included them in our qualitative analysis. Finally, 13 studies were included in qualitative synthesis and 11 studies were included in quantitative synthesis. The details of our study selection process are reported in Fig. 1.

### Study characteristics

The characteristics of the included trials are shown in Table 1. All of the included studies were single-center trials. A total of 381 participants with stroke were included in this review including 218 males and 163 females. The number of participants in each trial ranged from 12 to 44. The average age of participants was 59.3 years and the majority of studies originated from Korea and Europe, and two from China. Participants were inpatients in five trials [31, 34, 38, 42, 48], outpatients in two trials [47, 49], both inpatients and outpatients in one trial [40], and community-dwelling in one trial [32]. Four trials did not report the source of subjects [30, 33, 39, 41]. Inclusion and exclusion criteria of participants were reported in all of the included studies.

**Table 1:** Characteristics of included studies in this systematic review

Author,year	Mean age	Participents (M/F)	Intervention	Frenquency and duration of the hydrokinesitherapy	Outcomes/Measure(s)
Aidar 2018 [33]	52.2	36 (19/17)	EG: water-based aerobic exercise CG: no specific physical activity	45–60 min/day, 2 days/week, 12 weeks	Balance/BBS, TUGT, Getting up from a sitting position; Walking ability/Timed 7.62 m walk; Depression/Beck Depression Inventory (BDI)
Chan 2016 [47]	65.1	25 (13/12)	EG: water and land exercise CG: land-based physical therapy	60 min/day, 2 days/week, 6 weeks	Balance/BBS, TUGT, commnuity balance and mobility score; Walking ability/2 min walk test;
Chu 2004 [32]	62.5	12 (11/1)	EG: water-based exercise CG: arm exercise	60 min/day, 3 days/week, 8 weeks	Balance/BBS; Cardiorespiratory fitness/VO_2_max; Maximal workload; muscle strength; Gait speed;
Furnari 2014 [30]	70	40 (20/20)	EG: water-based exercise + conventional physical therapy CG: land-based physical therapy	60 min/day, 6 days/week, 8 weeks	ADL/BI, FIM, mRS; Spasticity/MAS; Baropodometric parameters;
Han 2017 [34]	60.9	20 (10/8)	EG: aquatic Treadmill exercise CG: land-based physical therapy	30 min/day, 5 days/week, 6 weeks	ADL/MBI; Cardiorespiratory fitness/VO_2_max; Walking ability/6 min walk test;
Kim 2016 [39]	68.6	20 (10/10)	EG: water-based exercise CG: land-based physical therapy	30 min/day, 5 days/week, 6 weeks	Balance/BBS, TUGT, FRT, Five Times Sit-to Stand Test; Walking ability/10-Meter Walk Test, FGA;
Lee 2018 [31]	60.5	37 (19/18)	EG: aquatic Treadmill exercise CG: land-based physical therapy	30 min/day, 5 days/week, 4 weeks	Balance/BBS; ADL/MBI; Isometric muscle strength/Knee extensor and flexor strength test; cardiorespiratory fitness/Symptom-limited exercise stress test; Motor function/FMA; QoL/EQ-5D index
Noh 2008 [38]	63.8	25 (11/14)	EG: Aichi and Halliwick exercise CG: land-based physical therapy	60 min/day, 3 days/week, 8 weeks	Balance/BBS; Weight-bearing ability; Gait ability/Modified Motor Assessment Scale gait scores; muscle strength;
Park 2011 [48]	53.8	44 (27/17)	EG: water-based exercise CG: land-based physical therapy	35 min/day, 6 days/week, 6 weeks	JPS;POMA;
Park 2016 [49]	43.8	28 (20/8)	EG: Halliwick exercise CG: land-based physical therapy	30 min/day, 3 days/week, 4 weeks	Walking speed; Walking cycle; Symmetry index of stance phase; Symmetry index of stride length; Affected side stride length; Affected side stance phase;
Tripp 2014 [42]	64.9	30 (19/11)	EG: Halliwick-Therapy + conventional physiotherapy CG: land-based physical therapy	45 min/day, 5 days/week, 2 weeks	Balance/BBS; functional gait ability/FAC; mobility/RMI
Zhang 2016 [41]	55.5	36 (17/19)	EG: aquatic Treadmill exercise CG: land-based physical therapy	40 min/day, 5 days/week, 8 weeks	ADL/BI; Spasticity/MAS; Surface electromyogram (EMG); dependence on gait/FAC
Zhu 2015 [40]	56.8	28 (22/6)	EG: aquatic Treadmill exercise CG: land-based physical therapy	45 min/day, 5 days/week, 4 weeks	Balance/BBS,TUGT,FRT; Walking ability/2-min walk test

*BBS*, Berg Balance scale; *TUGT*, Time Up to Go Test; *BDI*, Beck Depression Inventory; *ADL*, Activity of daiy living; *BI*, Barthel Index; *FIM*, Functional Independence Measure; *mRS*, modified Ranking Scale; *MAS*, Modified Ashworth scale; *MBI*, Modified Barthel Index; *FRT*, Functional reach test; *FGA*, Functional Gait Assessment; *FMA*, Fugl-meyer assessment; *QoL*, Quality of Life; *JPS*, joint position sense; *POMA*, performance-oriented mobility assessment; *FAC*, Functional Ambulation Category; *RMI*, Rivermead Mobility Index

The styles of hydrokinesitherapy employed were diverse. They included aquatic physical exercise [32, 33, 39, 47, 48], aquatic treadmill exercise [31, 34, 40, 41] and Halliwick [30, 38, 42, 49] performed in these trials. The frequency of hydrokinesitherapy were heterogeneous, with sessions ranging from 2 to 6 times per week, lasting between 30 and 60 min. The intervention duration ranged from 2 weeks to 12 weeks, 2 weeks in one trial [42], 4 weeks in three trials [31, 40, 49], 6 weeks in four trials [34, 39, 47, 48], 8 weeks in four trials [30, 32, 38, 41], and 12 weeks in one trial [33]. All studies included in this review carried out interventions without follow-up.

Of these 13 studies, eleven compared hydrokinesitherapy with land-based rehabilitation therapy [30, 31, 34, 38–42, 47–49], one with non-intervention (no specific physical activity) [33], one with arm exercise [32]. The age-predicted maximal heart rate (220-age) was used to monitor the intensity of exercise in three rails [31, 32, 34].

The Berg Balance Scale (BBS), Time Up and Go Test (TUGT), Functional Reach Test (FRT) (cm) were used to measure the balance ability. Walking speed, walk test such as 2 min walk test, 6 min walk test, 10 m walk test were used to measure the walking function. The Modified Barthel Index (MBI) and Barthel Index (BI) were used to measure ADL.

### Methodological quality of study

**Fig. 2:**
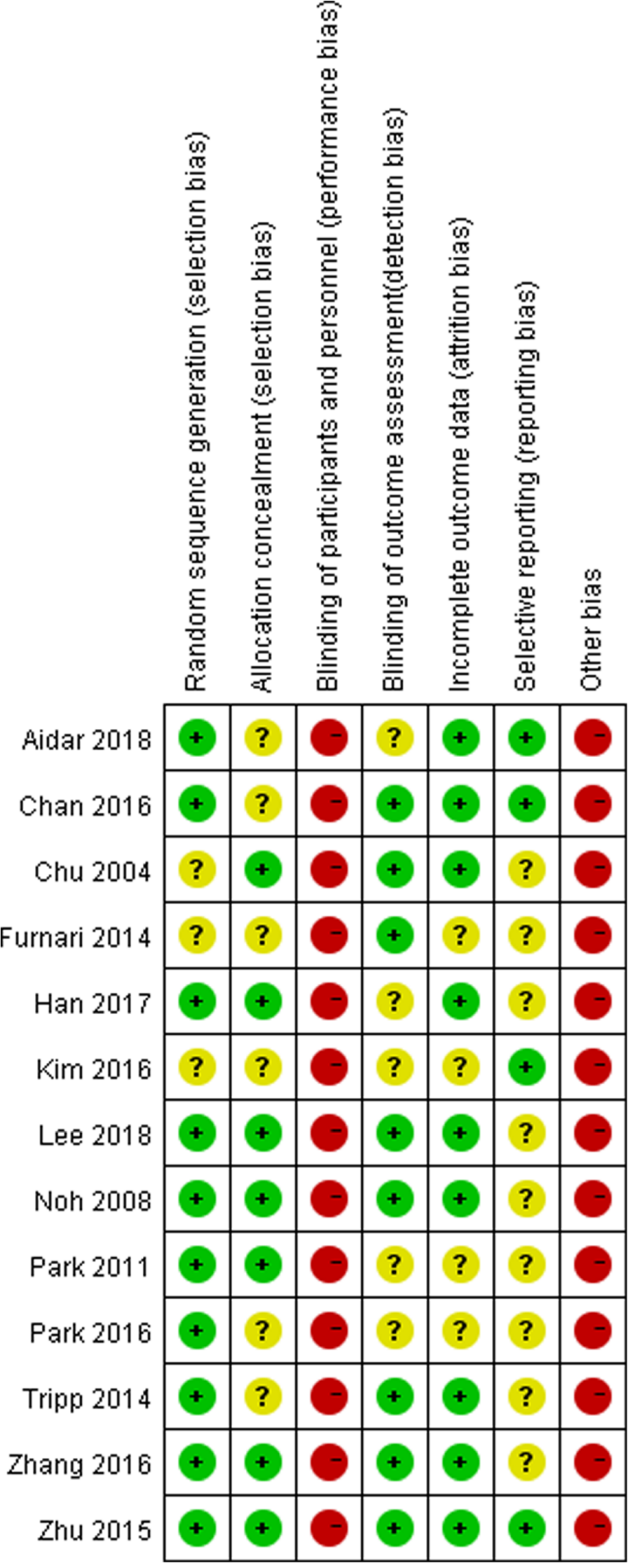
Risk of bias summary: review authors’ judgements about each risk of bias item for each included study

The risk of bias summary for included studies is shown in Fig. 2. Randomization allocation was reported in all included trials. Ten trials reported random sequence generation methods including using a random number generator [41], lottery [33], coin toss [47], sealed envelope technique [31, 34, 40, 42, 48], a blocked randomization procedure [38], drawing lots [41, 49]. Seven studies clearly reported allocation concealment [31, 32, 34, 38, 40, 41, 48]. The risk of performance bias was judged as “high risk” in all studies because patients and interventors could not be blinded in the hydrokinesitherapy program. Risk of detection bias of eight studies was considered to be low [30–32, 38, 40–42, 47]. The risk of attrition bias of nine studies was judged to be low because the research data were complete or the reasons of drop-out were reported [31–34, 38, 40–42, 47]. No study with missing data conducted an intention-to-treat analysis. Although no trials reported the clinical registration information and published protocols, four studies were judged as “low risk” in terms of selective reporting bias because all primary outcomes we focused on were reported in these trials [33, 39, 40, 47]. All studies were judged as “high risk” in terms of other bias due to their small sample size.

### Effects of intervention

#### Balance ability

**Fig. 3:**
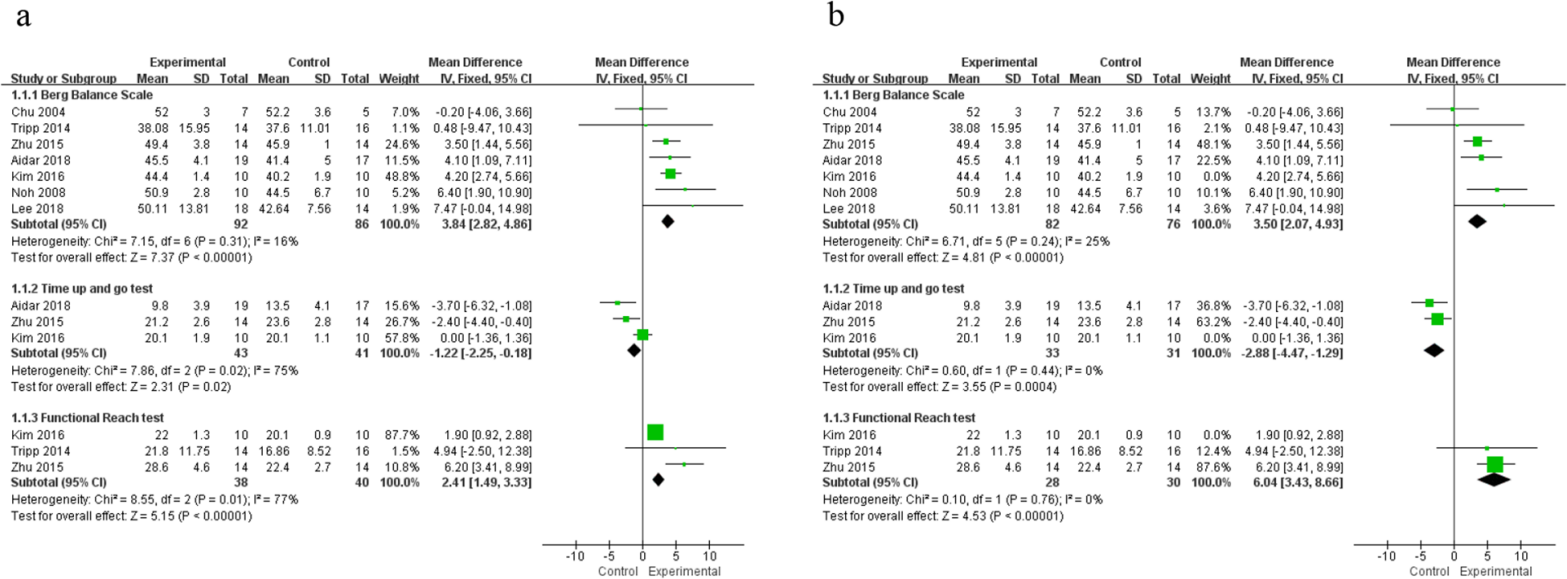
Meta-analysis (**a**) and sensitivity analysis (**b**) of hydrokinesitherapy on balance ability

As shown in Fig. 3a, seven studies [31–33, 38–40, 42] reported the effects of hydrokinesitherapy in terms of balance function in stroke survivors measured by using BBS, TUGT and FRT (cm). The subgroup analysis was performed because it was not appropriate to have an overall pooled analysis when different measure instruments were used among studies. The results demonstrated that hydrokinesitherapy can improve balance ability of stroke survivors by significantly increasing BBS scores (*n* = 178, MD = 3.84, 95% CI 2.82 to 4.86, *P* < 0.0001, I^2^ = 16%, the fixed-effect model). Three studies reported TUGT (*n* = 84, MD = − 1.22, 95% CI − 2.25 to − 0.18, *P* = 0.02, the fixed-effect model), with a substantial heterogeneity (I^2^ = 75%). Three studies reported FRT (*n* = 78, MD = 2.41, 95% CI 1.49 to 3.33, *P* < 0.0001, the fixed-effect model), with a substantial heterogeneity (I^2^ = 77%).

#### Walking ability

**Fig. 4:**
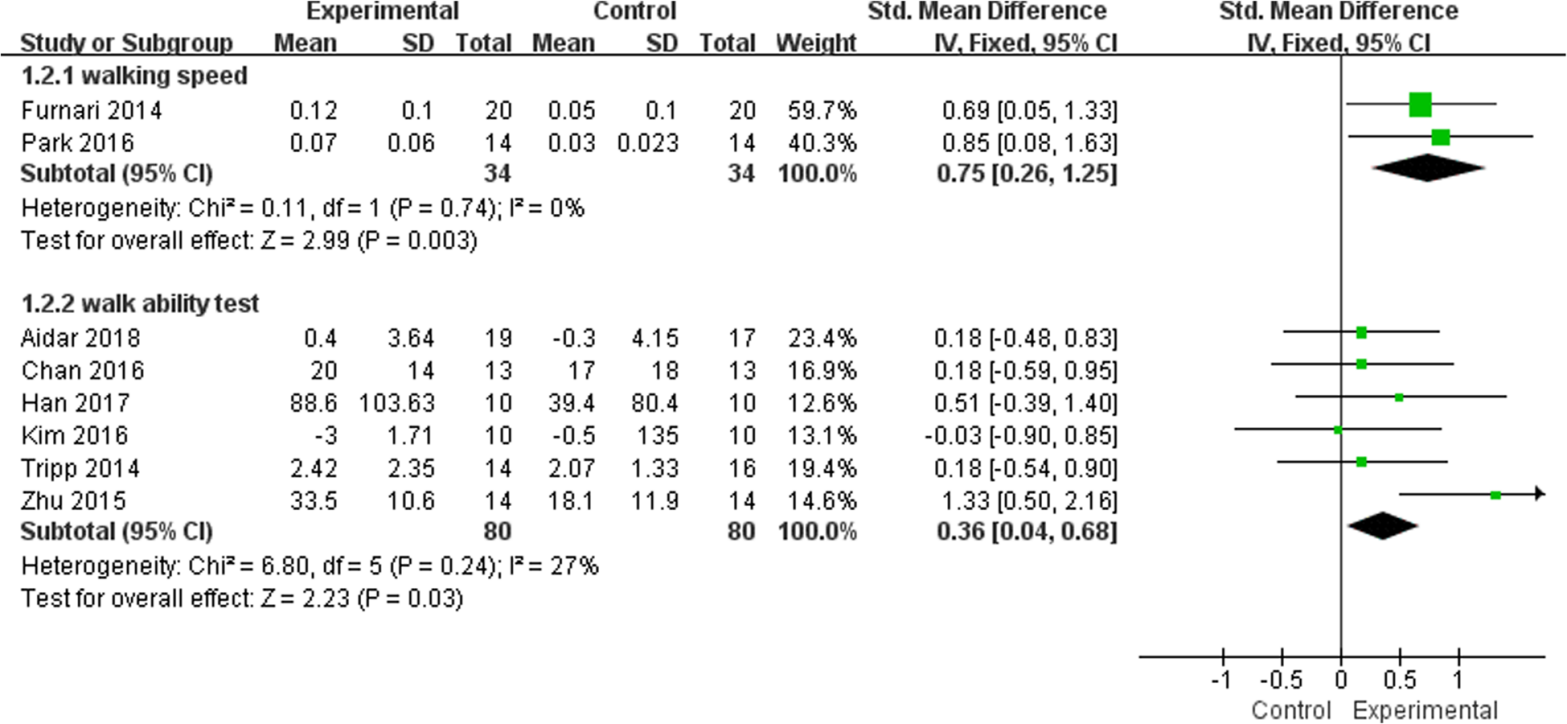
Meta-analysis of hydrokinesitherapy on walking ability

Six studies involving 198 patients reported the effects of hydrokinesitherapy on walking function measured by walking speed [30, 49], walking ability test such as 2 min walk test [40, 47], 6 min walk test [34], 7.62 m walk test [33], 10 m walk test [39]. Their results showed that the hydrokinesitherapy group had significantly augmented SMD of the walking speed compared with control group (*n* = 68, SMD = 0.75, 95% CI 0.26 to 1.25, *P* = 0.003, I^2^ = 0%, the fixed-effect model; Fig. 4). Two studies reported 2 min walk test [40, 47], one reported a 6 min walk test [34], one reported 7.62 m [33], one reported 10 m walk ability test [39]. The result showed that there was a significant difference between experimental group and control group in terms of walk ability test (*n* = 160, SMD = 0.36, 95% CI 0.04 to 0.68, *P* = 0.03, I^2^ = 27%, the fixed-effect model; Fig. 4).

#### Activity of daily life (ADL)

**Fig. 5:**
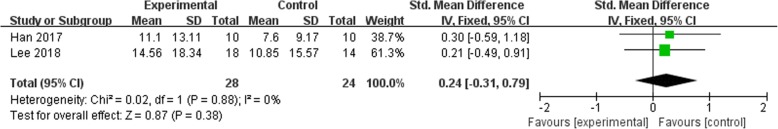
Meta-analysis of hydrokinesitherapy on ADL

Three studies examined the effects of hydrokinesitherapy on ADL using MBI or BI [31, 34, 41]. Two studies reported the MBI using Mean with SD [31, 34]. One study reported BI using range interquartile [41]. We contacted the original authors to request more data via email. But we did not receive any response. In order to ensure the reliability of results, only two studies included in the meta analysis for the ADL [31, 34]. The results indicated that there was no significant difference between hydrokinesitherapy group and control group (*n* = 52, SMD = 0.24, 95% CI − 0.31 to 0.79, *P* = 0.38, the fixed-effect model; Fig. 5).

#### Drop out

Among the included studies, seven trials reported the number and reasons of drop-out [31–33, 38, 41, 42, 47], two trials reported there were no drop out [34, 40], four studies did not report the drop out information [30, 39, 48, 49].

#### Adverse effects

There were no adverse events reported among the included trials.

#### Sensitivity analysis

In order to test the summary effect size, the random-effects model was used rather than the fixed-effects model. Differences were found for results of balance ability after changing the method. After excluding the study with the greatest weight in balance ability, the direction of outcome changed along with decreased heterogeneity (BBS: MD = 3.50, 95% CI 2.07 to 4.93, *P* < 0.00001, I^2^ = 25%; TUGT: MD = − 2.88, 95% CI − 4.47 to − 1.29, *P* = 0.0004, I^2^ = 0%; FRT: MD = 6.04, 95% CI 3.43 to 8.99, *P* < 0.00001, I^2^ = 0%; Fig. 3 b).

#### Publication bias

Due to the fact that the number of studies included for each outcome was less than 10, assessment of publication bias was not performed in this review because test power was too low to discern real asymmetry [50].

## Conclusions

This systematic review and meta-analysis collated evidence from RCTs assessing the clinical effectiveness of hydrokinesitherapy on equilibrium function, walking ability and ADL for stroke survivors. Thirteen RCTs were included in this review involving 381 patients. All of the included articles used short-term (≤ 3 months) intervention. Based on our meta-analysis, hydrokinesitherapy showed the potential to provide positive benefits in improving the equilibrium function and walking ability of stroke survivors compared with non-water exercise or no treatment. However, no significant improvement was found in terms of ADL. There were no adverse events reported in all of the included trials.

Due to inherent imprecision (limited sample size of the participants) and publication bias (the number of studies included was less than ten for each outcome), the level of evidence was downgraded. And there was no reason to upgrade the strength of evidence. Finally, the quality of the evidence was downgraded to ‘low-quality’ for the primary outcomes of balance ability and walking ability. Based on sensitivity analysis results, the trial reported by Kim et al. [39] had a significant impact on the results of the major pooled analysis. The reason we considered may be that the intervention in this studies was aquatic dual-task training in experimental group rather than only water-based exercise or water-based exercise plus land-based exercise without dual-task training [39].

### Strengths and limitations

The advantage of this review include its methodologically strong study design. First, a detailed search strategy was developed to obtain data by systematic searching different online electronic databases. Additionally, we developed and employed a structured study protocol to guide our search strategy, study selection, extraction of data and statistical analysis. Two reviewer authors independently screened, extracted, and evaluated the quality of data. Any disagreement was resolved by discussion and a final decision was determined by consultation with a third author. These methods reduced bias and transcription errors. Third, only RCTs were included in this review to ensure the design quality of the studies included. Finally, we clearly defined hydrokinesitherapy as a physical activity that is structured, designed, reproducible and implemented by a well-trained health care professional, rather than using a spa pool or bathtub to reduce the clinical heterogeneity.

However, there are also some limitations of this review. The first one we should note is that included trials demonstrated clinical heterogeneity in intervention test, intervention type, frequency and duration, which may result the evident heterogeneity. The type of hydrokinesitherapy used included aquatic physical therapy and aquatic treadmill exercise in the included trials. Both single hydrokinesitherapy, combinations of hydrokinesitherapy and land-based exercise were used in included trials. The intervention frequency ranged from 30 to 60 min per session and 2–6 times weekly. The intervention duration of the included studies ranged from 2 weeks to 12 weeks. Because of the variability in types of hydrokinesitherapy and measurement instruments of balance and walking ability, it was difficult to confirm a distinct relationship between specific types of hydrokinesitherapy and positive effect in balance function and walking ability.

Likewise, the instruments used to measure balance and walking ability in the included studies were heterogeneous. For the equilibrium function parameter, although 7 of the 13 included studies evaluated the effect of hydrokinesitherapy on balance ability and showed a significant difference between experimental group and control group. The variety of noncompatible measurement tools used (such as BBS, TUGT, FRT) results the difficulty to accurately calculate the effect sizes overall. For the walking function parameter, the same condition also existed. Few studies measured walk ability using the same measurement tools. Meanwhile, even though most of the measurement instruments, such as BBS, TUGT and FRT, had high validity and reliability, they were subjective which may result in the bias.

Additionally, it was impossible to blind participants and personnel in the hydrokinesitherapy program trial, thus performance bias is unavoidable. Another limitation was that the hydrokinesitherapy program used was of uniformly short duration without follow-up. Furthermore, methodological shortcomings, small sample size, a lack of reporting intention-to-treat analysis and limited of number of the included studies affected the credibility of our results. There were no adverse events reported in the included studies. However, we were greatly limited to determine the safety of practicing hydrokinesitherapy because of the poor and inconsistent reporting of adverse events. Therefore, caution is needed when interpreting the strength of the evidence.

### Clinical relevance and future directions

Compared to land-based exercise or no treatment, hydrokinesitherapy is more effective in improving the balance function and walking ability of stroke survivors. This meta-analysis suggested that hydrokinesitherapy could be a feasible intervention for stroke survivors. This review provides evidence for both clinicians and stroke survivors to recommend application of hydrokinesitherapy. Although we were not able to identify some of the ‘grey’ literature, it was unlikely that there would be a significant impact on our results. We provided a template for future studies that could be used to guide further research.

There are still some issues that deserve attention to in future research. Appropriate intervention intensity and frequency should be used in future studies on this topic such as the recommendations by the WHO [51] or the exercise training principles suggested by Campbell [52]. Intervention should be no less than 6 months with adequate follow-up periods if possible. Measurement instruments which are more sensitive and objective should be used in future studies (e.g. three-dimensional gait analysis and Pro-Kin system). In addition, other rehabilitation outcomes concerning hydrokinesitherapy for stroke survivors, including fall rates, quality of life and remission of pain, also deserve attention. Participant experience in water before stroke, such as fear of water, ability to swim, limited experience of water buoyancy, should be a focus on in future trials. Finally, and most importantly, when reporting trials, the CONSORT guidelines should be used to allow better evaluation of the methodological quality. There is a clear need for well-designed, multi-center, large-scale trials to evaluate safety and clinical effectiveness of hydrokinesitherapy for people with stroke.

In conclusion, hydrokinesitherapy may improve balance ability and walking ability in stroke survivors. However, due to the differences in the styles, durations and frequencies of hydrokinesitherapy training, and the small number of included studies with small sample size, caution is needed when interpreting these results. Well-designed, large-scale, multi-center RCTs with adequate follow-up periods and standardized training protocols are needed to provide more reliable evidence in terms of the effects of hydrokinesitherapy on balance and walking ability in post-stroke patients.

## Supplementary information

**Additional file 1.** Search strategy for each database.

## Data Availability

Not applicable.

## Abbreviations

ADL: Activity of daily living
BBS: Berg Balance Scale
DALYs: Disability-adjusted life-years
FRT: Functional Reach Test
RCTs: Randomized controlled trials
TUGT: Time Up and Go Test
