# Supplementary material for: Effects of hydrokinesitherapy on balance and walking ability in stroke survivors: a systematic review and meta-analysis of randomized controlled studies
Source: Eur Rev Aging Phys Act. 2019 Nov 13;16:21. doi: 10.1186/s11556-019-0227-0 (PMC6854709; doi:10.1186/s11556-019-0227-0)
Supplement: Supplementary file 1 — Additional file 1. Search strategy for each database. [file 11556_2019_227_MOESM1_ESM.docx]

[**Supplementary**](javascript:;) [**materials**](javascript:;)

Search strategy for each database

1. Medline via Pubmed

#1 “cerebrovascular disorders”[Mesh] or “brain injuries”[Mesh] or “brain injury, chronic”[Mesh]

#2 (“stroke$” or “cva” or “poststroke” or “post-stroke”)[tw]

#3 (“cerebrovasc$” or “cerebral vascular”)[tw]

#4 (“cerebral” or “cerebellar” or “brain$” or “vertebrobasilar”)[tw]

#5 (“infarct$” or “isch?emi$” or “thrombo$” or “emboli$” or “apoplexy”)[tw]

#6 #4 and #5

#7 (“cerebral” or “brain” or “subarachnoid”)[tw]

#8 (“haemorrhage” or “hemorrhage” or “haematoma” or “hematoma” or “bleed$”)[tw]

#9 #7 and #8

#10 “hemiplegia”[Mesh] or “paresis”[Mesh]

#11 (“hempar$” or “hemipleg$” or “brain injur$”)[tw]

#12 “Gait Disorders, Neurologic”[Mesh]

#13 #1 or #2 or #3 or #6 or #9 or #10 or #11 or #12

#14 “water”[Mesh] or “fresh water”[Mesh] or “seawater”[Mesh] or “hydrotherapy”[Mesh] or “swimming pools”[Mesh] or “swimming”[Mesh] or “balneology”[Mesh]

#15 (“water” or “water-based” or “seawater” or “aqua” or “aquatic$” or “hydrokinetic$” or “hydro-kinetic$” or “pool” or “pool-based” or “swimming pool”)[tw]

#16 #14 or #15

#17 “exercise”[Mesh] or “movement”[Mesh] or “locomotion”[Mesh] or “physical exertion”[Mesh] or “exercise therapy”[Mesh] or “physical endurance”[Mesh] or “physical fitness”[Mesh] or “sports”[Mesh] or “exercise movement techniques”[Mesh] or “fitness centers”[Mesh] or “physical therapy modalities”[Mesh] or “rehabilitation”[Mesh] or “gymnastics”[Mesh]

#18 #16 and #17

#19 ((“water” or “water-based” or “seawater” or “aqua” or “aquatic$” or “hydrokinetic$” or “hydro-kinetic$” or “pool” or “pool-based” or “swimming pool”) adj10 (“exercise$” or “fitness” or “physiotherap$” or “activit$” or “aerobic” or “training” or “therap$” or “rehabilitation” or “treadmill” or “walking” or “gymnastic$” or “calisthenic$”))[tw]

#20 (“treading water” or “swimming” or “swim” or “aquarobics” or “aquatone” or “Ai Chi” or “Halliwick” or “hydrotherap$” or “whirlpool bath$”)[tw]

#21 #18 or #19 or #20

#22 #13 and #21

#23 randomized controlled trial [pt]

#24 controlled clinical trial [pt]

#25 randomized [tiab]

#26 placebo [tiab]

#27 clinical trials as topic [mesh: noexp]

#28 randomly [tiab]

#29 trial [ti]

#30 #23 OR #24 OR #25 OR #26 OR #27 OR #28 OR #29

#31 animals [mh] NOT humans [mh]

#32 #30 NOT #31

#33 #22 AND #32

2. Embase via embase.com

#1 ‘cerebrovascular disease’/exp or ‘brain disease’/exp or ‘basal ganglion hemorrhage’/exp or ‘brain hemangioma’/exp or ‘brain hematoma’/exp or ‘brain hemorrhage’/exp or ‘brain infarction’/exp or ‘brain ischemia’/exp or ‘carotid artery disease’/exp or ‘cerebral artery disease’/exp or ‘cerebrovascular accident’/exp or ‘cerebrovascular malformation’/exp or ‘intracranial aneurysm’/exp or ‘occlusive cerebrovascular disease’/exp or ‘vertebrobasilar insufficiency’/exp

#2 (‘stroke*’ or ‘poststroke’ or ‘apoplex*’ or ‘cerebral vasc*’ or ‘brain vasc*’ or ‘cerebrovasc*’ or ‘cva*’ or ‘SAH’):ti:ab

#3 ((‘brain or cerebr$’ or ‘cerebell$’ or ‘vertebrobasil$’ or ‘hemispher$’ or ‘intracran$’ or ‘intracerebral’ or ‘infratentorial’ or ‘supratentorial’ or ‘middle cerebral artery’ or ‘MCA$’ or ‘anterior circulation’ or ‘posterior circulation’ or ‘basilar artery’ or ‘vertebral artery’ or ‘space-occupying’) adj5 (‘isch?emi$’ or ‘infarct$’ or ‘thrombo$’ or ‘emboli$’ or ‘occlus$’ or ‘hypoxi$’)):ti:ab

#4 ((‘brain$’ or ‘cerebr$’ or ‘cerebell$’ or ‘intracerebral’ or ‘intracran$’ or ‘parenchymal’ or ‘intraparenchymal’ or ‘intraventricular’ or ‘infratentorial’ or ‘supratentorial’ or ‘basal gangli$’ or ‘putaminal’ or ‘putamen’ or ‘posterior fossa’ or ‘hemispher$’ or ‘subarachnoid’) adj5 (‘h?emorrhag$’ or ‘h? ematoma$’ or ‘bleed$’)):ti:ab

#5 ‘hemiplegia’/exp or ‘paresis’/exp or ‘neurologic gait disorder’/exp

#6 (‘hemipleg$’ or ‘hemipar$’ or ‘paresis’ or ‘paraparesis’ or ‘paretic’):ti:ab

#7. or/#1-#6

#8 ‘water’/exp or ‘fresh water’/exp or ‘seawater’/exp or ‘hydrotherapy’/exp or ‘swimming pools’/exp or ‘swimming’/exp or ‘balneology’/exp

#9 (‘water’ or ‘water-based’ or ‘seawater’ or ‘aqua’ or ‘aquatic$’ or ‘hydrokinetic$’ or ‘hydrokinetic$’ or ‘pool’ or ‘pool-based’ or ‘swimming pool’):ti:ab

#10 #8 or #9

#11 ‘exercise’/exp or ‘movement’/exp or ‘locomotion’/exp or ‘physical exertion’/exp or ‘exercise therapy’/exp or ‘physical endurance’/exp or ‘physical fitness’/exp or ‘sports’/exp or ‘exercise movement techniques’/exp or ‘fitness centers’/exp or ‘physical therapy modalities’/exp or ‘rehabilitation’/exp or ‘gymnastics’/exp

#12 #10 and #11

#13 ((‘water’ or ‘water-based’ or ‘seawater’ or ‘aqua’ or ‘aquatic$’ or ‘hydrokinetic$’ or ‘hydrokinetic$’ or ‘pool’ or ‘pool-based’ or ‘swimming pool’) adj10 (‘exercise$’ or ‘fitness’ or ‘physiotherap$’ or ‘activit$’ or ‘aerobic’ or ‘training’ or ‘therap$’ or ‘rehabilitation’ or ‘treadmill’ or ‘walking’ or ‘gymnastic$’ or ‘calisthenic$’)):ti:ab

#14 (‘treading water’ or ‘swimming’ or ‘swim’ or ‘aquarobics’ or ‘aquatone’ or ‘Ai Chi’ or ‘Halliwick’ or ‘hydrotherap$’ or ‘whirlpool bath$’):ti:ab

#15 #12 or #13 or #14

#16 #7 and #15

#17 ‘randomized controlled trial’:ti,ab

#18 ‘controlled clinical trial’:ti,ab

#19 ‘randomized’:ti:ab

#20 ‘placebo’:ti:ab

#21 ‘clinical trials as topic’/exp

#22 ‘randomly’:ti:ab

#23 “trial”:ti

#24 #17 OR #18 OR #19 OR #20 OR #21 OR #22 OR #23

#25 animals /exp NOT humans /exp

#26 #24 NOT #25

#27 #16 AND#26

3. CINAHL via Ebsco

S1 (MH “Cerebrovascular Disorders+”) or (MH “stroke patients”) or (MH “stroke units”)

S2 TI ( stroke or poststroke or post-stroke or cerebrovasc* or cerebral vasc or cva) or AB ( stroke or poststroke or post-stroke or cerebrovasc* or cerebral vasc or cva)

S3 TI ( brain* or cerebr* or cerebell* or vertebrobasilar ) or AB ( brain* or cerebr* or cerebell* or vertebrobasilar )

S4 TI ( ischemi* or ischaemi* or infarct* or thrombo* or emboli* or apoplexy* ) or AB ( ischemi* or ischaemi* or infarct* or thrombo* or emboli* or apoplexy* )

S5 S3 and S4

S6 TI ( brain* or cerebr* or cerebell* or subarachnoid ) or AB ( brain* or cerebr* or cerebell* or subarachnoid )

S7 TI ( haemorrhage* or hemorrhage* or haematoma* or hematoma* or bleed* ) or AB ( haemorrhage* or hemorrhage* or haematoma* or hematoma* or bleed* )

S8 S6 and S7

S9 (MH “Hemiplegia”)

S10 TI ( hemipleg* or hemipar* or paresis or paretic or brain injur* ) or AB ( hemipleg* or hemipar* or paresis or paretic or brain injur* )

S11 (MH “Brain Injuries”)

S12 S1 or S2 or S5 or S8 or S9 or S10 or S11

S13 (MH“water+”) or (MH“fresh water+”) or (MH“seawater+”) )or (MH“hydrotherapy+”) )or (MH “swimming pools+”) or(MH “swimming+”) or(MH “balneology+”)

S14 TI (water or water-based or seawater or aqua or aquatic$ or hydrokinetic$ or hydro-kinetic$ or pool or pool-based or swimming pool)

S15 S13 or S14

S16 (MH“exercise+”) or (MH“movement+”) or (MH“locomotion+”) or (MH “physical exertion+”) or (MH “exercise therapy+”) or (MH “physical endurance+”) or (MH “physical fitness+”) or (MH “sports+”) or (MH “exercise movement techniques+”) or (MH “fitness centers+”) or (MH “physical therapy modalities+”) or (MH “rehabilitation+”) or (MH “gymnastics+”)

S17 S15 and S16

S18 TI (water or water-based or seawater or aqua or aquatic* or hydrokinetic* or hydro-kinetic* or pool or pool-based or swimming pool) adj10 TI(exercise* or fitness or physiotherap* or activit* or aerobic or training or therap* or rehabilitation or treadmill or walking or gymnastic* or calisthenic*)

S19 TI (treading water or swimming or swim or aquarobics or aquatone or Ai Chi or Halliwick or hydrotherap* or whirlpool bath*)

S20 S17 or S18 or S19

S21 S12 and S20

S22 PT(randomized controlled trial)

S23 PT (controlled clinical trial)

S24TI(randomized)

S25TI(placebo)

S26 (MH “clinical trials as topic+”)

S27 TI(randomly)

S28 TI (trial)

S29 S22 OR S23 OR S24 OR S25 OR S26 OR S27 OR S28

S30 limit to human

S31 S21 AND S29 AND S30

4. SPORTDiscus via Ebsco

S1 (KW “Cerebrovascular Disorders+”) or (KW “stroke patients”) or (KW “stroke units”)

S2 TI ( stroke or poststroke or post-stroke or cerebrovasc* or cerebral vasc or cva) or AB ( stroke or poststroke or post-stroke or cerebrovasc* or cerebral vasc or cva)

S3 TI ( brain* or cerebr* or cerebell* or vertebrobasilar ) or AB ( brain* or cerebr* or cerebell* or vertebrobasilar )

S4 TI ( ischemi* or ischaemi* or infarct* or thrombo* or emboli* or apoplexy* ) or AB ( ischemi* or ischaemi* or infarct* or thrombo* or emboli* or apoplexy* )

S5 S3 and S4

S6 TI ( brain* or cerebr* or cerebell* or subarachnoid ) or AB ( brain* or cerebr* or cerebell* or subarachnoid )

S7 TI ( haemorrhage* or hemorrhage* or haematoma* or hematoma* or bleed* ) or AB ( haemorrhage* or hemorrhage* or haematoma* or hematoma* or bleed* )

S8 S6 and S7

S9 (KW “Hemiplegia”)

S10 TI ( hemipleg* or hemipar* or paresis or paretic or brain injur* ) or AB ( hemipleg* or hemipar* or paresis or paretic or brain injur* )

S11 (KW “Brain Injuries”)

S12 S1 or S2 or S5 or S8 or S9 or S10 or S11

S13 (KW“water*”) or (KW“fresh water*”) or (KW“seawater*”) )or (KW“hydrotherapy*”) )or (KW “swimming pools*”) or(KW “swimming*”) or(KW “balneology*”)

S14 TI (water or water-based or seawater or aqua or aquatic$ or hydrokinetic$ or hydro-kinetic$ or pool or pool-based or swimming pool)

S15 S13 or S14

S16 (KW“exercise*”) or (KW“movement*”) or (KW“locomotion*”) or (KW “physical exertion*”) or (KW “exercise therapy*”) or (KW “physical endurance*”) or (KW “physical fitness*”) or (KW “sports*”) or (KW “exercise movement techniques*”) or (KW “fitness centers*”) or (KW “physical therapy modalities*”) or (KW “rehabilitation*”) or (KW “gymnastics*”)

S17 S15 and S16

S18 TI (water or water-based or seawater or aqua or aquatic* or hydrokinetic* or hydro-kinetic* or pool or pool-based or swimming pool) adj10 TI(exercise* or fitness or physiotherap* or activit* or aerobic or training or therap* or rehabilitation or treadmill or walking or gymnastic* or calisthenic*)

S19 TI (treading water or swimming or swim or aquarobics or aquatone or Ai Chi or Halliwick or hydrotherap* or whirlpool bath*)

S20 S17 or S18 or S19

S21 S12 and S20

S22 TI(randomized controlled trial) or AB(randomized controlled trial)

S23 TI (controlled clinical trial) or AB (controlled clinical trial)

S24 TI(randomized) or AB(randomized)

S25 TI(placebo) or AB(placebo)

S26 (KW “clinical trials as topic*”)

S27 TI(randomly) or AB(randomly)

S28 TI (trial) or AB(trial)

S29 S22 OR S23 OR S24 OR S25 OR S26 OR S27 OR S28

S30 SU human

S31 S21 AND S29 AND S30

5. CENTRAL

#1 [mh ^“cerebrovascular disorders”] or [mh “basal ganglia cerebrovascular disease”] or [mh “brain ischemia”] or [mh “carotid artery diseases”] or [mh “intracranial arterial diseases”] or [mh “intracranial arteriovenous malformations”] or [mh “intracranial embolism and thrombosis”] or [mh “intracranial hemorrhages”] or [mh ^“stroke”] or [mh “brain infarction”] or [mh ^“stroke, lacunar”] or [mh ^“vasospasm, intracranial”] or [mh ^“vertebral artery dissection”] or [mh ^“brain injuries”] or [mh ^“brain injury, chronic”]

#2 (stroke or poststroke or post-stroke or cerebrovasc* or “brain next vasc*” or “cerebral next vasc*” or cva* or apoplex* or SAH):ti,ab,kw(Word variations have been searched)

#3 ((brain* or cerebr* or cerebell* or intracran* or intracerebral) near/5 (isch*emi* or infarct* or thrombo* or emboli* or occlus*)):ti,ab,kw (Word variations have been searched)

#4 ((brain* or cerebr* or cerebell* or intracerebral or intracranial or subarachnoid) near/5 (haemorrhage* or hemorrhage* or haematoma* or hematoma* or bleed*)):ti,ab,kw (Word variations have been searched)

#5 [mh ^hemiplegia] or [mh paresis] or [mh “gait disorders, neurologic”]

#6 (hempar* or hemipleg* or brain next injur*):ti,ab,kw (Word variations have been searched)

#7 {or #1-#6}

#8 [mh ^water] or [mh ^“fresh water”] or [mh ^seawater] or [mh ^hydrotherapy] or [mh ^“swimming pools”] or [mh ^swimming] or [mh ^balneology]

#9 (water or “water-based” or “seawater” or “aqua” or “aquatic$” or “hydrokinetic$” or “hydrokinetic$” or “pool” or “pool-based” or “swimming pool”):ti,ab,kw

#10 #8 or #9

#11 [mh exercise] or [mh movement] or [mh locomotion] or [mh “physical exertion”] or [mh “exercise therapy”] or [mh “physical endurance”] or [mh “physical fitness”] or [mh sports] or [mh “exercise movement techniques”] or [mh “fitness centers”] or [mh “physical therapy modalities”] or [mh rehabilitation] or [mh gymnastics]

#12 #10 and #11

#13 ((water or water-based or seawater or aqua or aquatic$ or hydrokinetic$ or hydro-kinetic$ or pool or pool-based or “swimming pool”) adj10 (exercise$ or fitness or physiotherap$ or activit$ or aerobic or training or therap$ or rehabilitation or treadmill or walking or gymnastic$ or calisthenic$)):ti,ab,kw

#14 (“treading water” or swimming or swim or aquarobics or aquatone or “Ai Chi” or Halliwick or hydrotherap$ or “whirlpool bath$”):ti,ab,kw

#15 #12 or #13 or #14

#16 #7 and #15

#17 randomized controlled trial:pt

#18 controlled clinical trial:pt

#19 randomized:ti,ab

#20 placebo:ti,ab

#22 [mh “clinical trials as topic”]

#22 randomly:ti,ab

#23 trial:ti

#24 {or #17-#23}

#25 [mh animals] NOT [mh humans]

#26 #24 NOT #25

#27 #16 AND #26
